# Diseases of the Urinary and Male Sexual Organs

**Published:** 1885-06

**Authors:** 


DISEASES OF THE URINARY AND MALE SEXUAL ORGANS. By William T. Belfield, M. D., author of Relations of Micro-Organisms to Disease (Cartwright Lectures, 1883); Pathologist to the Cook County Hospital; Surgeon to the Genito-Urinary Department Central Dispensary, Chicago; Physician to the Oakwood Retreat, Geneva, Wisconsin; Professor of Microscopy, Chicago College of Dental Surgery. New York: William Wood & Co. 1884.

This is the October number of Wood's Library of Popular Authors for 1884.

The author, in his preface, says: When the preparation of this volume was undertaken, it was the author's hope to present a resumé of current knowledge of the topics discussed, with comments suggested by personal observation and experience.

He has done his work well and has given to the profession one of the most interesting and instructive books we have read in a long time.

